# DL- and PO-phosphatidylcholines as a promising learning and memory enhancer

**DOI:** 10.1186/1476-511X-10-25

**Published:** 2011-01-28

**Authors:** Tetsu Nagata, Takahiro Yaguchi, Tomoyuki Nishizaki

**Affiliations:** 1 Division of Bioinformation, Department of Physiology, Hyogo College of Medicine, 1-1 Mukogawa-cho, Nishinomiya 663-8501, Japan

## Abstract

In the water maze test, oral administration with 1,2-dilynoleoyl-*sn*-glycero-3-phosphocholine (DLPhtCho)(5 mg/kg) alone or DLPhtCho (5 mg/kg) plus 1-palmitoyl-2-oleoyl-*sn*-glycero-3-phosphocholine (POPhtCho)(5 mg/kg) significantly shortened the prolonged acquisition latency for rats intraperitoneally injected with scopolamine, with more efficient effect than (POPhtCho)(5 mg/kg) alone, arachidonic acid (AA)(5 mg/kg) alone, docosahexaenoic acid (DHA)(5 mg/kg) alone, or 1-palmitoyl-2-linoleil-*sn*-glycero-3-phosphoserine (PLPhtSer)(5 mg/kg) alone. POPhtCho (5 mg/kg) alone or DLPhtCho (5 mg/kg) plus POPhtCho (5 mg/kg) also significantly shortened the prolonged retention latency for rats intraperitoneally injected with scopolamine, but otherwise no significant effect was obtained with DLPhtCho (5 mg/kg) alone, AA (5 mg/kg) alone, DHA (5 mg/kg) alone, or PLPhtSer (5 mg/kg) alone. Oral co-administration with DLPhtCho (5 mg/kg) and POPhtCho (5 mg/kg) significantly shortened the acquisition latency for rats untreated with scopolamine as compared with the latency for administration with polyethylene glycol (PEG), DLPhtCho alone at doses of 5 and 10 mg/kg, or POPhtCho alone at doses of 5 and 10 mg/kg, while no efficient effect on the retention latency was obtained. To assess the effect of DLPhtCho and POPhtCho on cognitive functions for humans, Mini Mental State Examination (MMSE) test was performed in subjects with cognitive disorders (the average MMSE score, 15). Oral co-intake with DLPhtCho (50 mg) and POPhtCho (45 mg) once after breakfast everyday raised the score to over 20, corresponding to normal cognitive functions, throughout 5 months after intake, and the increase in the score was significantly greater than that for oral intake with DLPhtCho (100 mg/day) alone or POPhtCho (90 mg/kg) alone. Taken together, the results of the present study show that co-intake with DLPhtCho and POPhtCho could enhance learning and memory ability and improve cognitive disorders for both the animals and humans with a promising efficacy.

## Introduction

Accumulating evidence has pointed to a variety of lipids as a regulatory mediator of cognitive functions. *Cis*-unsaturated free fatty acids such as AA, oleic, linoleic, linolenic, and DHA, that are produced by phospholipase A_2_-catalyzed hydrolysis of PhtCho [1], are recognized to serve as a retrograde messenger for expression of long-term potentiation (LTP), a cellular model of learning and memory [2]. Those free fatty acids facilitate hippocampal synaptic transmission by targeting presynaptic nicotinic ACh receptors under the influence of protein kinase C (PKC) [3,4]. This accounts for contribution of the free fatty acids to LTP formation [5,6]. *Cis*-unsaturated free fatty acids, accordingly, might enhance learning and memory ability.

In our earlier studies, DLPhtCho potentiated α7 nicotinic ACh receptor responses and facilitated hippocampal synaptic transmission in an α7 nicotinic ACh receptor-dependent manner [7]. POPhtCho, alternatively, improved scopolamine-induced impairment of spatial learning and memory for rats, possibly by enhancing long-term depression (LTD), another cellular model of learning and memory, in concert with decreased expression of the α-amino-3-hydroxy-5-methyl-4-isoxazole propionic acid (AMPA) receptor subunit GluR1 on the plasma membrane [8]. Notably, POPhtCho ameliorated cognitive decline for humans [8]. PhtChos, thus, could exert its beneficial action against learning and memory disorders. In addition, phosphatidylserine (PhtSer) has been also suggested to serve as a cognitive enhancer, although its effect is still under discussion [9,10].

The present study was conducted to assess the efficacy of DLPhtCho, POPhtCho, AA, DHA, and PLPhtSer on learning and memory functions. We show here that co-intake with DLPhtCho and POPhtCho enhances spatial learning and memory ability and improves cognitive disorders for both the animals and humans, with more beneficial effect than each alone, AA, DHA, or PLPhtSer alone.

## Materials and methods

### Animal care

All procedures have been approved by the Animal Care and Use Committee at Hyogo College of Medicine and were in compliance with the National Institutes of Health Guide for the Care and Use of Laboratory Animals.

### Participants

The study for human was approved by Institutional Review Board at Hyogo College of Medicine and informed consent was obtained from all the participants.

### Water maze test

A circular plastic water tank with 180 cm in diameter and 45 cm in deep was used for a water maze test. The entire inside of the pool was painted black, and the pool was filled up to 25 cm from the bottom with muddy water containing India ink at 22°C. A platform (11 cm in diameter) painted black was placed into water, the top sinking 1 cm below water surface. The pool was put in a test room, where there were several marks that rats were able to see from the pool. The position of the marks remained unchanged throughout testing. A platform was located in the constant position, i.e., in the middle of one quadrant, equidistant from the center and edge of the pool. Wistar rats (male, 7 weeks) facing the wall of the pool were placed into water at one of 5 positions selected at random, and time from start to escape onto the platform (acquisition latency) was measured. When succeeded, rats were allowed to stay on the platform for 10 s. Scopolamine (1 mg/kg) was intraperitoneally injected 30 min before water maze task as a model of spatial learning and memory disorders. DLPhtCho, POPhtCho, AA, DHA, and PLPhtSer dissolved with PEG (final volume, 0.1 ml) or PEG alone (final volume, 0.1 ml) alone was orally administered to rats everyday throughout experiments from 7 days prior to the beginning of water maze test. Water maze task was performed two trials per day, and the second trial began 2 min after the end of the first trial. Rats received the task for consecutive 8 days and the acquisition latency (time form start to arrival onto the plate) was measured.

Eight days later, the platform was removed and the retention latency (time from the start to arrival to the place where the platform had been set, 30 s in maximum) was measured.

### MMSE test

MMSE test was performed on 310 subjects (135 males and 175 females) ranging in age from 59 to 95 years (average, 76 ± 1.2 years old), who had age-related cognitive disorders before and after oral intake with DLPhtCho (100 mg/day), extracted from soybean lecithin, for 21 subjects, POPhtCho (90 mg/day), extracted from egg lecithin, for 214 subjects, and DLPhtCho (50 mg/day) plus POPhtCho (45 mg/day) for 75 subjects. Full marks are 30, and less than 20 corresponds to mild cognitive impairment and dementia.

### Statistical analysis

Statistical analysis was carried out using Fisher's Protected Least Significant Difference (PLSD) test, Dunnett's test, and paired *t*-test.

## Results and discussion

**Figure 1:**
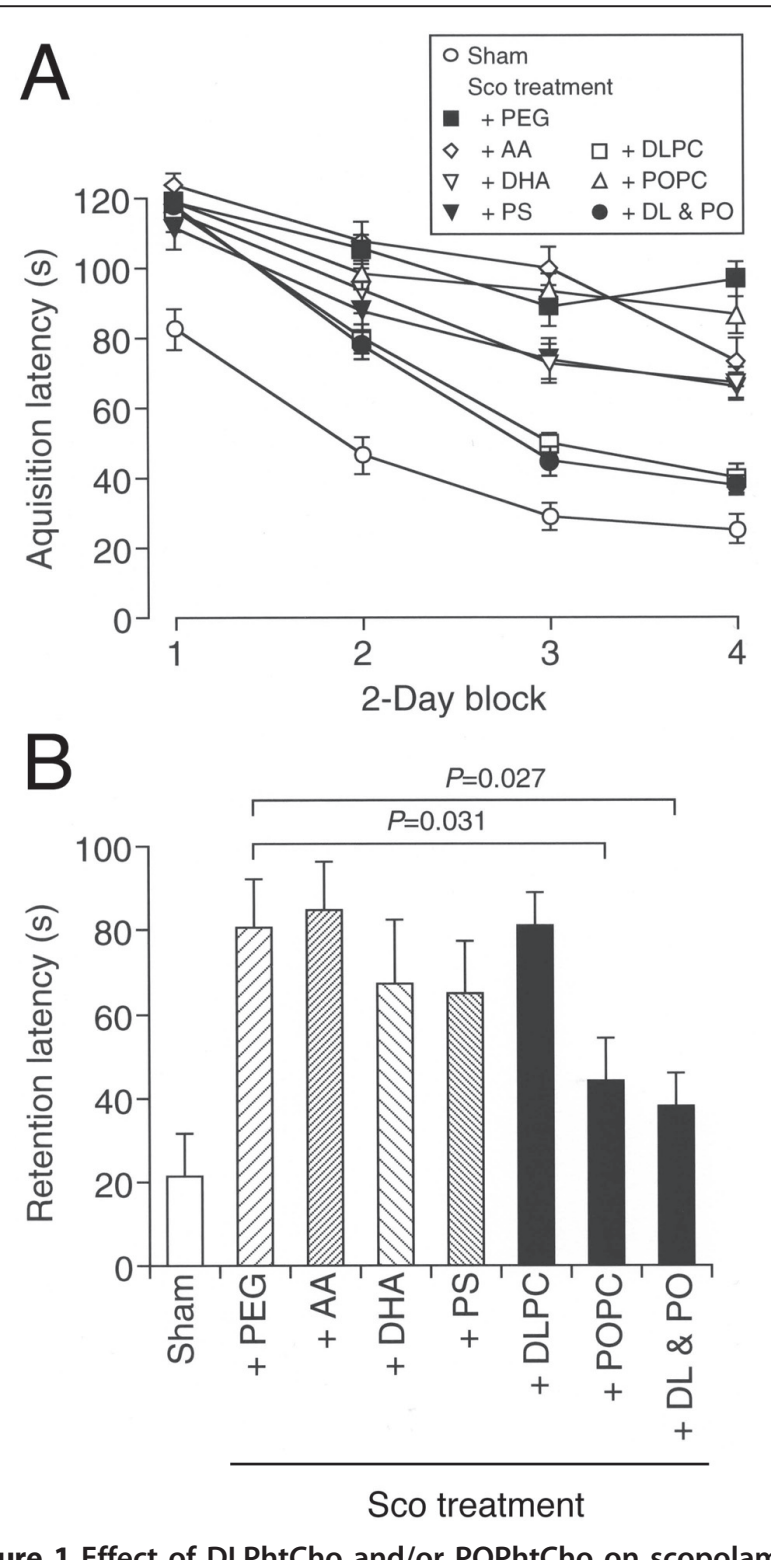
**Effect of DLPhtCho and/or POPhtCho on scopolamine-induced impairment of spatial learning and memory**. Rats were orally administered with PEG, AA, DHA, PLPhtSer (PS), DLPhtCho (DLPC), POPhtCho (POPC), or DLPhtCho plus POPhtCho (DL & PO) at a dose of 5 mg/kg everyday throughout experiments from 7 days prior to the beginning of water maze test. Saline or scopolamine (1 mg/kg) was intraperitoneally injected 30 min prior to water maze task. (**A**) Each point represents the mean (± SEM) acquisition latency from consecutive 2 days (n = 6). (**B**) Each column represents the mean (± SEM) retention latency (n = 6). *P *values, Dunnett's test. Sham, intraperitoneal injection with saline and oral administration with PEG; Sco treatment + PEG, intraperitoneal injection with scopolamine and oral administration with PEG; Sco treatment + AA, intraperitoneal injection with scopolamine and oral administration with AA; Sco treatment + DHA, intraperitoneal injection with scopolamine and oral administration with DHA; Sco treatment + PS, intraperitoneal injection with scopolamine and oral administration with PLPhtSer; Sco treatment + DLPC, intraperitoneal injection with scopolamine and oral administration with DLPhtCho; Sco treatment + POPC, intraperitoneal injection with scopolamine and oral administration with POPhtCho; Sco treatment + DL & PO, intraperitoneal injection with scopolamine and oral administration with DLPhtCho plus POPhtCho.

In the water maze test, intraperitoneal injection with scopolamine markedly prolonged the acquisition latency (Figure 1A), indicating scopolamine-induced impairment of spatial learning. Oral administration with DLPhtCho (5 mg/kg) alone or DLPhtCho (5 mg/kg) plus POPhtCho (5 mg/kg) significantly shortened the prolonged acquisition latency (*P *< 0.001 as compared with the latency for scopolamine-treated rats with oral administration with PEG, Fisher's PLSD test), each reaching similar levels for sham groups, while no significant effect was obtained with POPhtCho (5 mg/kg) alone (Figure 1A). Oral administration with DHA (5 mg/kg) or PLPhtSer (5 mg/kg) also shortened the prolonged latency, but the effect was not significant (Figure 1A). No efficient effect was obtained with AA (5 mg/kg); conversely, AA more prolonged the acquisition latency (Figure 1A), suggesting that AA might worsen spatial learning deficits.

Scopolamine also prolonged the retention latency (Figure 1B), indicating scopolamine-induced impairment of spatial memory. Notably, POPhtCho (5 mg/kg) alone or DLPhtCho (5 mg/kg) plus POPhtCho (5 mg/kg) significantly shortened the prolonged retention latency, although DLPhtCho (5 mg/kg) alone had no effect on it (Figure 1B). No efficient effect was obtained with AA (5 mg/kg), DHA (5 mg/kg) or PLPhtSer (5 mg/kg)(Figure 1B).

*Cis*-unsaturated free fatty acids such as AA and DHA, systemically applied, are promptly metabolized and decomposed before arriving in the brain. This may account for no/less effect of AA or DHA against spatial learning and memory deficits, even though evidence has pointed to those lipids as a cognitive enhancer in the *in vitro *systems. PLPhtSer here shortened the prolonged acquisition latency for rats treated with scopolamine, but to much lesser extent as compared with the extent for DLPhtCho alone or DLPhtCho plus POPhtCho, and the lipid had no effect on the prolonged retention latency. This may support the note for less efficient effect of PhtSer on memory or other cognitive functions [10].

In our earlier study, DLPhtCho facilitated hippocampal synaptic transmission by enhancing α7 ACh receptor responses [7], but POPhtCho, otherwise, enhanced LTD by reducing expression of AMPA receptor subunit GluR1 on the plasma membrane [8]. This suggests different actions on learning and memory functions between DLPhtCho and POPhtCho. In support of this note, DLPhtCho improved the prolonged acquisition latency without affecting the prolonged retention latency and POPhtCho ameliorated the prolonged retention latency without affecting the prolonged acquisition latency, and DLPhtCho plus POPhtCho exerted both the actions.

**Figure 2:**
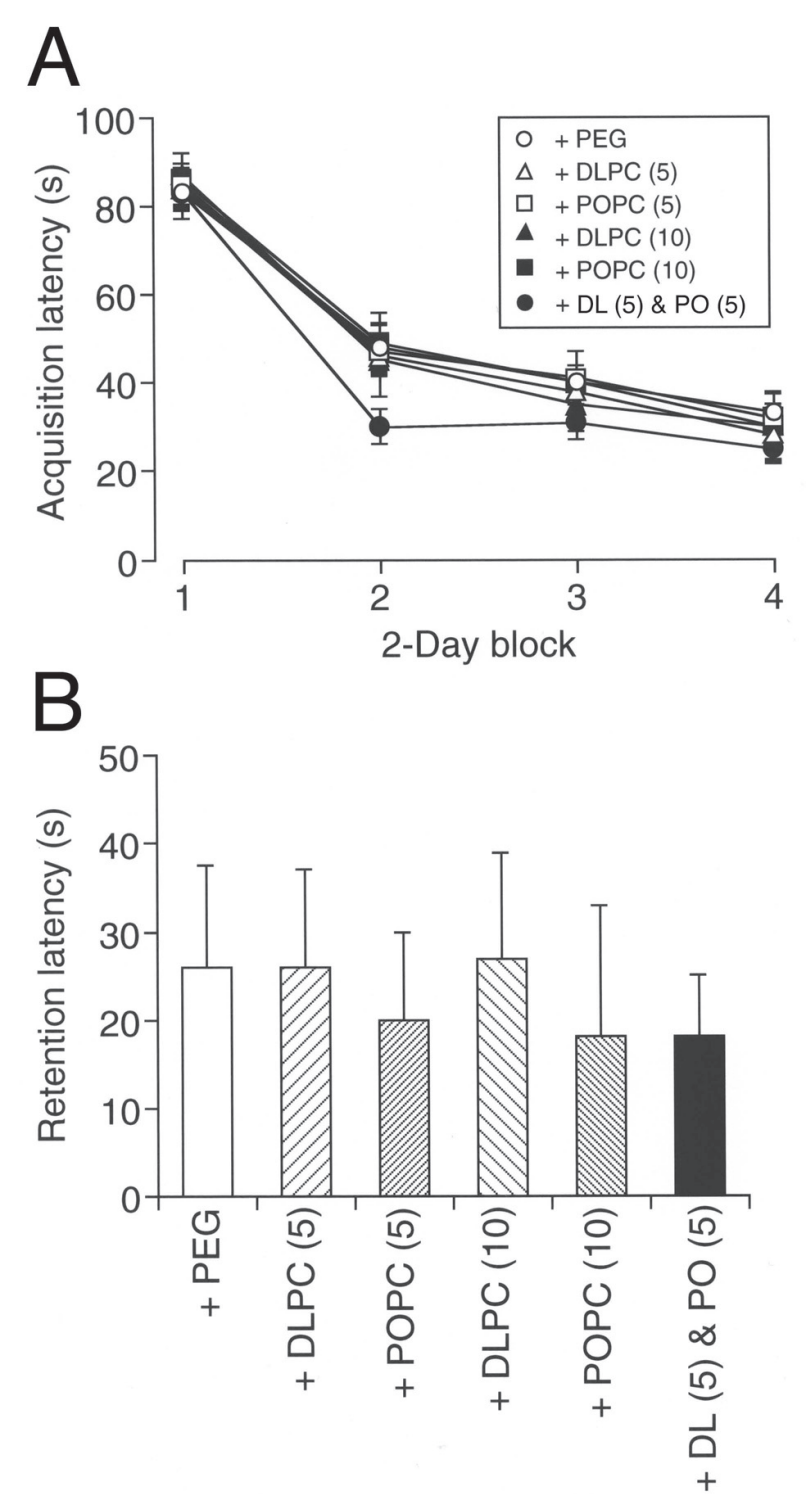
**Effect of DLPhtCho and/or POPhtCho on spatial learning and memory for normal rats**. Rats were orally administered with PEG, DLPhtCho (DLPC), POPhtCho (POPC), or DLPhtCho plus POPhtCho (DL & PO) everyday throughout experiments from 7 days prior to the beginning of water maze test. (**A**) Each point represents the mean (± SEM) acquisition latency from consecutive 2 days (n = 6). (**B**) Each column represents the mean (± SEM) retention latency (n = 6). + PEG, oral administration with PEG; + DLPC (5), oral administration with DLPhtCho (5 mg/kg); + DLPC (10), oral administration with DLPhtCho (10 mg/kg); + POPC (5), oral administration with POPhtCho (5 mg/kg); + POPC (10), oral administration with POPhtCho (10 mg/kg); + DL (5) & PO (5), oral administration with DLPhtCho (5 mg/kg) plus POPhtCho (5 mg/kg).

Amazingly, oral co-administration with DLPhtCho (5 mg/kg) and POPhtCho (5 mg/kg) significantly shortened the acquisition latency for rats untreated with scopolamine as compared with the latency for administration with PEG, DLPhtCho alone at doses of 5 and 10 mg/kg, or POPhtCho alone at doses of 5 and 10 mg/kg (*P *< 0.001, Fisher's PLSD test)(Figure 2A). Oral administration with POPhtCho alone at doses of 5 and 10 mg/kg or DLPhtCho (5 mg/kg) plus POPhtCho (5 mg/kg) also shortened the retention latency for untreated rats, but no significant effect was obtained (Figure 2B). It is indicated from these results that DLPhtCho and/or POPhtCho could serve as an enhancer of learning and memory for normal rats.

PhtCho, systemically applied and conveyed in the brain, is hydrolyzed into *cis*-unsaturated free fatty acid and lysophosphatidylcholine by phospholipase A_2 _or into choline and phosphatidic acid by phospholipase D, followed by further hydrolysis into *cis*-unsaturated free fatty acid and lysophosphatidic acid by phospholipase A_2_. As previously shown, *cis*-unsaturated free fatty acid, lysophosphatidylcholine, and lysophosphatidic acid persistently potentiate nicotinic ACh receptor responses [3,11,12], to induce an LTP-like long-lasting facilitation of hippocampal synaptic transmission [4,13] or LTP [5,6]. In addition, choline activates α7 nicotinic ACh receptors as a selective agonist [14], thereby stimulating the cholinergic systems closely related to cognitive functions. PhtCho metabolites as well as unhydrolyzed PhtCho by itself, accordingly, could enhance learning and memory ability or ameliorate learning and memory impairment.

**Figure 3:**
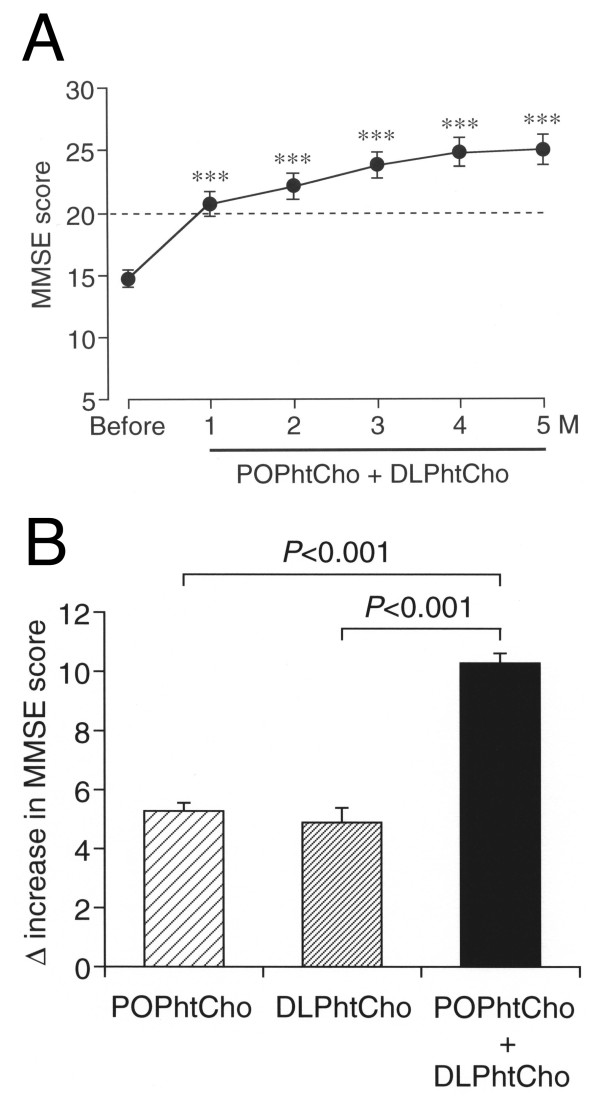
**Effect of DLPhtCho and/or POPhtCho on cognitive disorders for humans**. MMSE test was carried out once a month before and after oral intake with DLPhtCho, POPhtCho, and DLPhtCho plus POPhtCho once after breakfast every day. (**A**) Each point represents the mean (± SEM) MMSE score at the periods as indicated for 75 subjects with oral intake with DLPhtCho (50 mg/day) plus POPhtCho (45 mg/kg). **P < 0.001 as compared with the score before oral intake, paired *t*-test. (**B**) An MMSE score rise was calculated 5 months after oral intake with DLPhtCho (100 mg/day) alone for 21 subjects, POPhtCho (90 mg/day) alone for 214 subjects, and DLPhtCho (50 mg/day) plus POPhtCho (45 mg/kg) for 75 subjects. Each column represents the mean (± SEM) MMSE rise. *P *values, Dunnett's test.

We finally examined the effect of DLPhtCho and/or POPhtCho on cognitive disorders for humans. Of all the subjects (310) tested here approximately 65% belonged in mild cognitive impairment and dementia. For 75 subjects with oral intake with DLPhtCho and POPhtCho, the average MMSE score before intake was 14.7 ± 0.7 (Figure 3A). Oral co-intake with DLPhtCho (50 mg/day) and POPhtCho (45 mg/day) once after breakfast everyday significantly raised MMSE scores, the average score reaching over 20, i.e., normal cognitive functions, throughout 5 months after intake (Figure 3A). An MMSE score rise 5 months after co-intake with DLPhtCho (50 mg/day) and POPhtCho (45 mg/day) was significantly greater than the rise 5 months after DLPhtCho (100 mg/day) alone and POPhtCho (90 mg/day) alone (Figure 3B). These results indicate that co-intake with DLPhtCho and POPhtCho is capable of ameliorating mild cognitive impairment and dementia for humans, with more efficient effect than each alone. This also suggests that continuous intake with DLPhtCho and POPhtCho from a diet or dietary supplements could prevent dementia.

In conclusion, the results of the present study show that co-intake with DLPhtCho and POPhtCho improves spatial learning and memory impairment for rats treated with scopolamine, enhances spatial learning and memory ability for normal rats, and ameliorates mild cognitive impairment and dementia for humans, with more beneficial effect than each alone. DLPhtCho and POPhtCho, thus, could prevent and improve dementia with a promising efficacy.

## List of abbreviations

AA: arachidonic acid; AMPA: α-amino-3-hydroxy-5-methyl-4-isoxazole propionic acid; PhtCho: phosphatidylcholine; DHA: docosahexaenoic acid; DLPhtCho: 1,2-dilynoleoyl-*sn*-glycero-3-phosphocholine; LTD: long-term depression; LTP: long-term potentiation; MMSE: Mini Mental State Examination; PhtSer: phosphatidylserine; PEG: polyethylene glycol; PKC: protein kinase C; POPhtCho: 1-palmitoyl-2-oleoyl-*sn*-glycero-3-phosphocholine; PLPhtSer: 1-palmitoyl-2-linoleil-*sn*-glycero-3-phosphoserine; PLSD: Protected Least Significant Difference.

## Competing interests

The authors declare that they have no competing interests.

## Authors' contributions

TN and TY designed the study, performed all the experiments, and analyzed the data. TN supervised and coordinated the study and prepared the manuscript. All authors read and approved the final manuscript.
