# Erratum to: DL- and PO-phosphatidylcholines as a promising learning and memory enhancer

**DOI:** 10.1186/s12944-015-0120-4

**Published:** 2015-09-24

**Authors:** Tetsu Nagata, Takahiro Yaguchi, Tomoyuki Nishizaki

**Affiliations:** Division of Bioinformation, Department of Physiology, Hyogo College of Medicine, 1-1 Mukogawa-cho, Nishinomiya, 663-8501 Japan

## Erratum

The original version of this article unfortunately contained a mistake. Section “Materials and methods” subsection “participants” was incorrect in the HTML and PDF versions of this article. The original article stated: “Participants The study for human was approved by Institutional Review Board at Hyogo College of Medicine and informed consent was obtained from all the participants”, this should read: “Participants The study for human was performed in Tsuna Hospital (Hyogo, Japan) and informed consent was obtained from all the participants.”
